# Supplementary material for: Using Radiomics-Based Machine Learning to Create Targeted Test Sets to Improve Specific Mammography Reader Cohort Performance: A Feasibility Study
Source: J Pers Med. 2023 May 24;13(6):888. doi: 10.3390/jpm13060888 (PMC10300999; doi:10.3390/jpm13060888)
Supplement: Supplementary file 1 [file jpm-13-00888-s001.zip › jpm-2275384-supplementary.pdf]

Supplementary Table S1. Details of the radiomic features.

**Feature type**		**Feature name**
First-order (*n*=28)		Mean, standard deviation, skewness, kurtosis, minimum, maximum,<br>intensity percentile (the 5th , 10th , 15th , 20th , 25th , 50th , 75th , 80th , 85th , 90th , and 95th) ,<br>intensity interquatile range (100-0, 99-1, and 95-5),<br>mean*, variance*, median*, mode*, skewness*, kurtosis*, energy*, entropy*
Textural (*n*=159)	Gray level co-occurrence matrix (GLCM)	Autocorrelation, contrast, correlation1, correlation2, cluster prominence, cluster shade, dissimilarity, enegy, entropy, homogeneity1, homogeneity2, maximum likelihood, sum of squares of variance, sum average, sum variance, sum entropy, difference variance, information measures of correlation1, information measures of correlation2, inverse difference normalized, inverse difference moment normalized<br><br>Orientation = 0°, 45°, 90°, 135° (averaging along four directions)<br>Pixel distance = 1, 3, 5, 9<br>Number of levels = 64
	Gray level run length matrix (GLRLM)	Short runs emphasis, long runs emphasis, grey level nonuniformity, run percentage, run length nonuniformity, low grey-level run emphasis, high gray-level run emphasis<br><br>Offset = 1, 2, 3, 4
	Gray level difference statistics (GLDM)	Homogeneity, contrast, energy, entropy, mean<br><br>Orientation = 0°, 45°, 90° , 135° (averaging along four directions)
	Neighborhood gray tone difference matrix (NGTDM)	Coarseness, contrast, busyness, complexity, strength<br><br>Neighborhood size = 3, 5, 9 pixels
	Statistical feature matrix	Coarseness measure, contrast measure, periodicity measure, roughness measure<br><br>Neighborhood size = 4, 8 pixels
	Gray-level sharpness measure (GLSM)	Gradient, laplacian, wavelet decomposition (level 2 and 3 wavelet decomposition, db6 wavelet)
	Laws texture energy measures	Texture energy from LL kernel, texture energy from EE kernel, texture energy from SS kernel, average texture energy from LE and EL kernels, average texture energy from ES and SE kernels, average texture energy from LS ans SL kernels<br><br>Filter size = 3, 5, 7 pixels
	Fractional dimension texture analysis	Roughness

Transform -based (*n*=16)	Gabor texture features	Wavelengh = 4<br>Orientation = 0 °, 30 °, 60 °, 90 °, 120 °, 150 °
	RFS filter based features	Filter size = 49 pixels<br>Scale = 1,2,4<br>Orientation = 0 °, 30 °, 60 °, 90 °, 120 °, 150 °
	Fourier transform spectrum	Radial sum, angular sum

*Features were extracted from histogram of the median-filtered image.

Supplementary Table S2. Comparisons of AUCs between cohort A and cohort B using 2000-time stratified bootstrap in detecting diagnostic errors.

**Error types**	**Normalization**	**ROIs**	**p values**
false positive error	Yes	square	0.242
		RA	0.211
		whole	0.158
	No	square	0.246
		RA	0.872
		whole	**0.024**
false negative error	Yes	square	0.308
		RA	0.750
		whole	0.076
	No	square	0.703
		RA	0.968
		whole	0.419
false location error	Yes	square	0.709
		RA	0.891
		whole	0.183
	No	square	0.203
		RA	0.580
		whole	0.598

Statistically significant p values are in bold.

Supplementary Table S3. The effect of ROI placement on error detection for readers from cohort A and cohort B.

Error types	Radiologist	Normalization	ROIs	p values	95%CI
False positive error	Cohort A	Yes	square vs. RA	0.493	-0.146 ~ 0.070
			RA vs. whole	0.903	-0.135 ~ 0.152
			square vs. whole	0.594	-0.135 ~ 0.077
		No	square vs. RA	0.206	-0.066 ~ 0.306
			RA vs. whole	0.191	-0.300 ~ 0.060
			square vs. whole	1.000	-0.150 ~ 0.150
	Cohort B	Yes	square vs. RA	0.175	-0.332 ~ 0.060
			RA vs. whole	0.940	-0.173 ~ 0.187
			square vs. whole	0.272	-0.359 ~ 0.101
		No	square vs. RA	0.474	-0.212 ~ 0.099
			RA vs. whole	0.264	-0.097 ~ 0.355
			square vs. whole	0.529	-0.152 ~ 0.296
False negative error	Cohort A	Yes	square vs. RA	0.341	-0.526 ~ 0.182
			RA vs. whole	0.779	-0.420 ~ 0.561
			square vs. whole	0.726	-0.669 ~ 0.465
		No	square vs. RA	0.445	-0.196 ~ 0.446
			RA vs. whole	0.225	-0.470 ~ 0.110
			square vs. whole	0.717	-0.350 ~ 0.241
	Cohort B	Yes	square vs. RA	0.818	-0.332 ~ 0.262
			RA vs. whole	0.743	-0.224 ~ 0.314
			square vs. whole	0.948	-0.292 ~ 0.312
		No	square vs. RA	0.606	-0.154 ~ 0.264
			RA vs. whole	0.553	-0.215 ~ 0.115
			square vs. whole	0.961	-0.196 ~ 0.206
False location error	Cohort A	Yes	square vs. RA	0.295	-0.703 ~ 0.213
			RA vs. whole	0.119	-0.089 ~ 0.783
			square vs. whole	0.546	-0.229 ~ 0.433
		No	square vs. RA	0.905	-0.354 ~ 0.313
			RA vs. whole	0.671	-0.287 ~ 0.184
			square vs. whole	0.743	-0.499 ~ 0.356
	Cohort B	Yes	square vs. RA	0.357	-0.579 ~ 0.209

			RA vs. whole	0.601	-0.237 ~ 0.410
			square vs. whole	0.642	-0.516 ~ 0.318
		No	square vs. RA	0.465	-0.198 ~ 0.432
			RA vs. whole	0.862	-0.456 ~ 0.382
			square vs. whole	0.606	-0.225 ~ 0.385

Supplementary Table S4. The effect of normalization on error detection for readers from cohort A and cohort B.

Error types	Radiologist	ROIs	p values	95%CI
false positive error	Cohort A	square	0.554	-0.220 ~ 0.118
		RA	0.267	-0.082 ~ 0.295
		whole	0.745	-0.156 ~ 0.112
	Cohort B	square	0.304	-0.358 ~ 0.112
		RA	0.676	-0.252 ~ 0.163
		whole	0.464	-0.130 ~ 0.285
false negative error	Cohort A	square	0.105	-0.639 ~ 0.060
		RA	0.967	-0.360 ~ 0.376
		whole	0.264	-0.667 ~ 0.183
	Cohort B	square	0.869	-0.387 ~ 0.327
		RA	0.636	-0.189 ~ 0.309
		whole	0.726	-0.231 ~ 0.161
false location error	Cohort A	square	0.518	-0.248 ~ 0.493
		RA	0.119	-0.089 ~ 0.783
		whole	0.699	-0.309 ~ 0.207
	Cohort B	square	0.776	-0.438 ~ 0.327
		RA	0.213	-0.142 ~ 0.635
		whole	0.429	-0.182 ~ 0.429
